# WW-Domain Containing Protein Roles in Breast Tumorigenesis

**DOI:** 10.3389/fonc.2018.00580

**Published:** 2018-12-13

**Authors:** Abrar Jamous, Zaidoun Salah

**Affiliations:** Al Quds-Bard College for Arts and Sciences, Al Quds University, Abu Dis, Palestine

**Keywords:** WW domain, WWOX, hippo pathway, E3 ligases, breast cancer

## Abstract

Protein-protein interactions are key factors in executing protein function. These interactions are mediated through different protein domains or modules. An important domain found in many different types of proteins is WW domain. WW domain-containing proteins were shown to be involved in many human diseases including cancer. Some of these proteins function as either tumor suppressor genes or oncogenes, while others show dual identity. Some of these proteins act on their own and alter the function(s) of specific or multiple proteins implicated in cancer, others interact with their partners to compose WW domain modular pathway. In this review, we discuss the role of WW domain-containing proteins in breast tumorigenesis. We give examples of specific WW domain containing proteins that play roles in breast tumorigenesis and explain the mechanisms through which these proteins lead to breast cancer initiation and progression. We discuss also the possibility of using these proteins as biomarkers or therapeutic targets.

## Introduction

Protein function is determined by the domains and motifs that it harbors. These domains can be functional domains such as the catalytic domains found in many enzymes, or structural domains that are important for protein-protein interactions or the assembly of multi-protein complexes. Thus, protein's function is determined by its catalytic activity and its partners. One of these important domains found in many proteins is the WW domain. WW domain, is the smallest naturally occurring module. It consists of ~35–40 amino acids, including two highly conserved tryptophans (W), after which the module is named (WW). The two Ws are separated by 20–23 amino acids (1). Based on their ligand preference, WW domains are classified into five different classes. WW domains are very important domains that are involved in very critical cellular processes including; transcription, splicing, ubiquitination, apoptosis, cell growth, proliferation, and differentiation (2). Because of its link to many critical cellular processes and its widespread distribution among many proteins, WW module is linked to many diseases including; Liddle's syndrome of hypertension, muscular dystrophy, Alzheimer's and Huntington's diseases, and cancer (1, 2).

## WWOX and Breast Tumorigenesis

WW domain oxidoreductase (WWOX) is a tumor suppressor gene, which is altered in different types of cancer, including breast cancer (3). In breast cancer, WWOX gene is lost even in pre-invasive stages (4, 5). Moreover, recent studies have shown a correlation between WWOX expression and the clinical outcome of breast cancer (6–10).

In addition to clinical data that supports WWOX role in breast tumorigenesis, different animal models have proved its function in proper mammary gland development and tumorigenesis. It was shown that WWOX is highly expressed in mammary gland tissue and that its targeted deletion results in mammary gland fibroadenoma (11), as well as impaired mammary gland development (12, 13). Moreover, it was shown that aging WWOX-heterozygous C3H knockout mice develop higher incidence of mammary tumors. These tumors bear altered gene expression that resembles altered gene expression in human breast cancer (14).

WWOX mediates its functions by interacting with and modulating the activities of different proteins either through its WW domains or in a WW domain independent manner. Nonetheless, WWOX has also an oxidoreductase domain that seems to play a role in tumorigenesis too. WWOX alters different cellular processes involved in tumorigenesis. As discussed below, these cell activities include apoptosis, genomic instability, metabolism and metastasis.

### WWOX Function in Breast Cancer Cell Apoptosis

**Figure 1:**
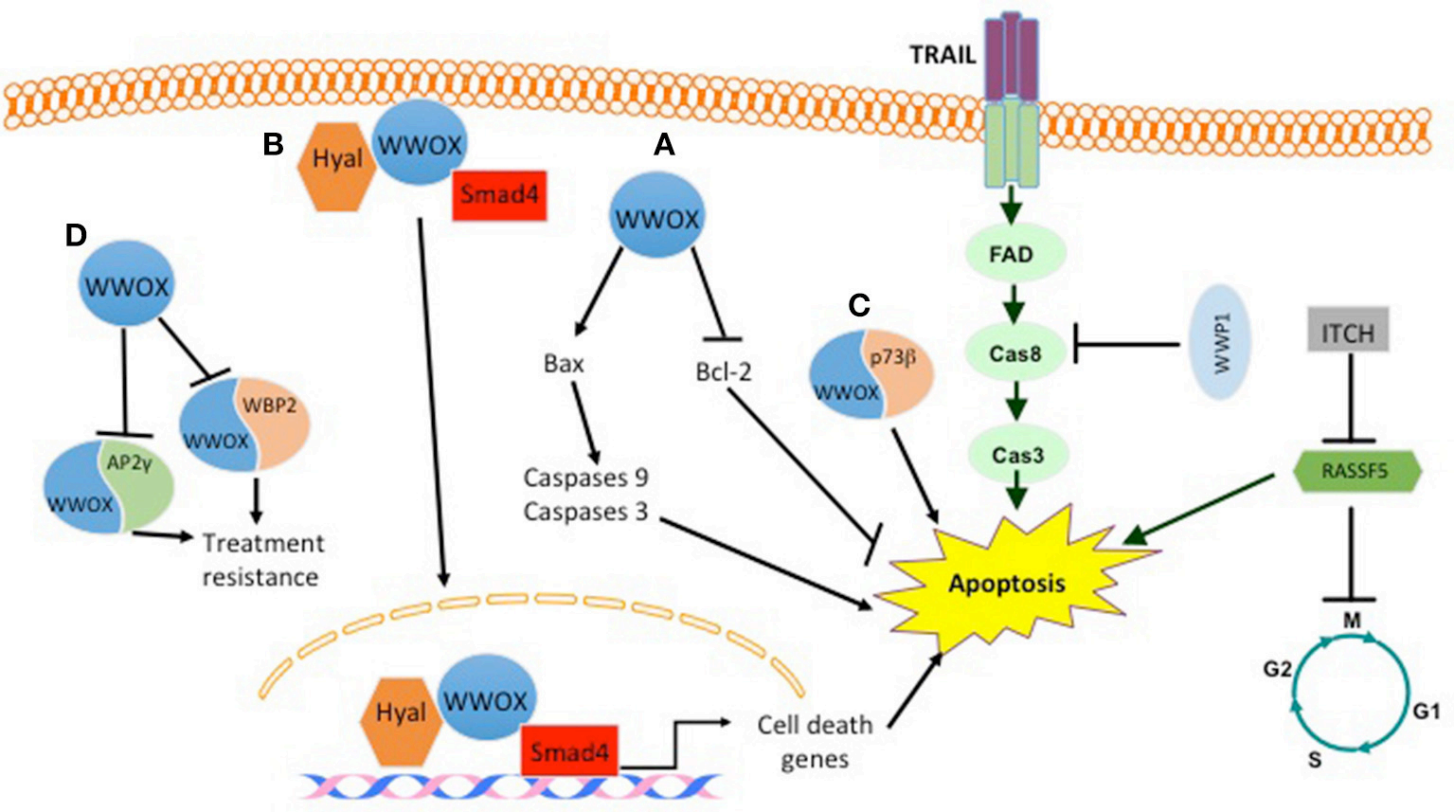
Effect of some WW domain proteins on cell death. WWOX induces cell death by **(A)** inducing BAX levels and decreasing BCL2 levels. **(B)** Upon hylauronan treatment WWOX complexes with hyaluronidase and Smad4. This complex translocates to the nucleus and induces the expression of Smad4 pro-apoptotic target genes. **(C)** WWOX binds to P73b and induces its cytoplasmic dependent cell death. **(D)** WWOX binds and sequesters WBP2 and AP2γ in the cytoplasm. This inhibits their treatment resistance phenotype. E3 ligases also manipulate breast cancer cell death. WWP1 inhibits TRAIL mediated cell death. ITCH by inhibiting RASSF5 prevents apoptosis and activates cell cycle.

Different published findings have linked WWOX to apoptosis. It was revealed that WWOX overexpression or endogenous WWOX reactivation in breast cancer cells leads to apoptotic cell death *in vitro* and suppression of breast tumor growth *in vivo*. These effects are mediated by reducing the expression levels of the anti-apoptotic protein Bcl-2 and inducing the pro-apoptotic protein BCL2-associated X protein (Bax) (15) (Figure 1). Furthermore, WWOX was shown to interact with p73β in MCF7 breast cancer cell line (Figure 1). This interaction, although was not tested in breast cancer cells, leads to a cytoplasmic p73β dependent cell death (16). WWOX was also shown to interact and stabilize p53 and confer cellular sensitivity to apoptotic stress (17). Another study demonstrated that WWOX induces breast cancer cell apoptosis by triggering Smad4 transcriptional activity (Figure 1) (18). In the clinical context of cell death and apoptosis, WWOX was shown to interact with two very important factors involved in hormone treatment resistance. These factors include activating protein 2γ (AP2γ) and WW domain-binding protein 2(WBP2) (Figure 1). WWOX interacts with AP2γ and sequesters it in the cytoplasm and inhibits its transactivational function (19). For WBP2, WWOX physically interacts with WBP2 and suppresses ER transactivation pathways (Figure 1) (20).

### WWOX and Genomic Instability in Breast Cancer

**Figure 2:**
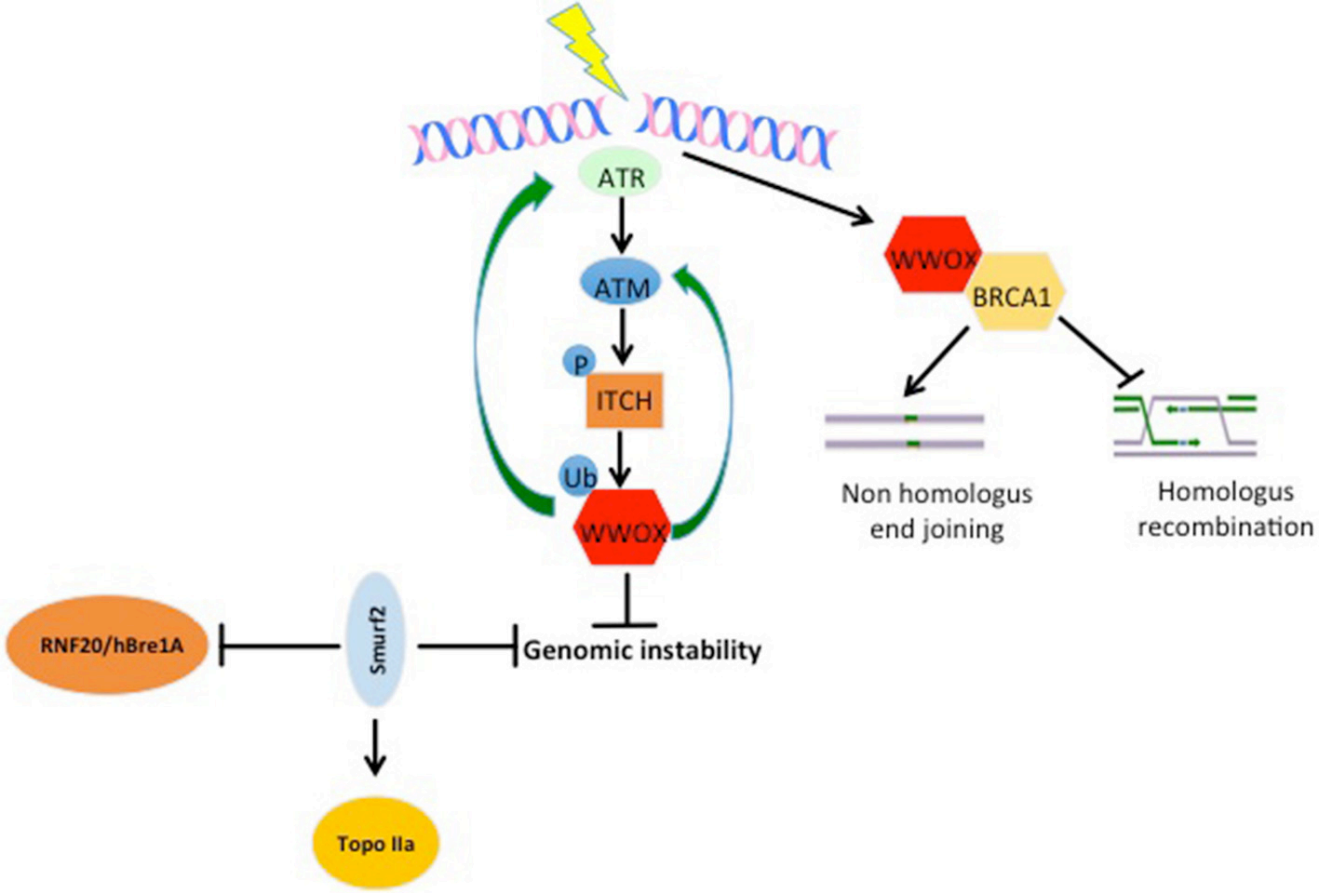
WW domain protein roles in genomic stability. Upon DNA damage, ATR and ATM get activated. When activated, they can phosphorylate and activate the E3 ligase ITCH. Active ITCH can further ubiquitinate and activate WWOX leading to its translocation to the nucleus. In the nucleus, WWOX, in a positive feedback loop activates ATM and ATR and enhances DNA damage response and repair. Moreover, WWOX can interact with BRCA1 and enhance Non-homologous end joining over homologus recombination repair pathway. E3s also play roles in genomic stability too. SMURF2 increase genomic stability by inhibiting the activity of RNF20/hBre1A, the major ubH2B-specific E3 and by stabilizing topoisomerase IIa (Topo IIa). ITCH enhances genomic integrity by increasing the efficiency of DNA damage repair (DDR). Upon DNA damage ITCH is activated by ATM. Active ITCH can further ubiquitinate and activate WWOX leading to its translocation to the nucleus. In the nucleus, WWOX, in a positive feedback loop, activates ATM and ATR and enhances DNA damage response and repair.

Genomic instability is one of the important cancer hallmarks involved in tumor initiation as well as tumor progression (21, 22). WWOX was recently assigned an important function in DNA damage response (DDR) (23). Abu-Odeh et al. demonstrated that WWOX interacts with Ataxia telangiectasia mutated (ATM) and activates both ATM and Ataxia telangiectasia mutated RAD3-related (ATR) following DNA damage. They also showed that WWOX knockdown is associated with less activation of DDR signaling molecules, and consequently less efficient DNA repair and thus more genomic instability (Figure 2) (23, 24). In the context of response and resistance of cells to DNA damaging therapies, a recent study found that WWOX deficiency increases survival of cells after ionizing radiation-induced double strand breaks (DSBs), and that WWOX restoration in MDA MB231 breast cancer cells, that lack endogenous WWOX, leads to reduced survival upon gamma irradiation. On the contrary, WWOX-silenced cells survived bleomycin treatments as compared to WWOX-expressing MCF10A cells (25). Moreover, in this study, WWOX interaction with Breast Cancer gene1 (BRCA1) was shown to affect DNA DSB repair pathway choice and thus affects cell response to DSB inducing agents (25, 26). These findings prove that WWOX plays an important role in DDR and genomic stability, which might make it a marker for the success of DDR-targeting biological therapies. Since WWOX seems to play a very important role in DDR, and that its loss impairs the repair process and leads to genomic instability, it will be interesting to see whether WWOX loss would enhance the activity of PARP inhibitors. Here, based on PARP inhibitors mechanism of action, it is expected that cells that lack WWOX will be more sensitive to this type of therapy.

### WWOX and Cancer Metabolism

**Figure 3:**
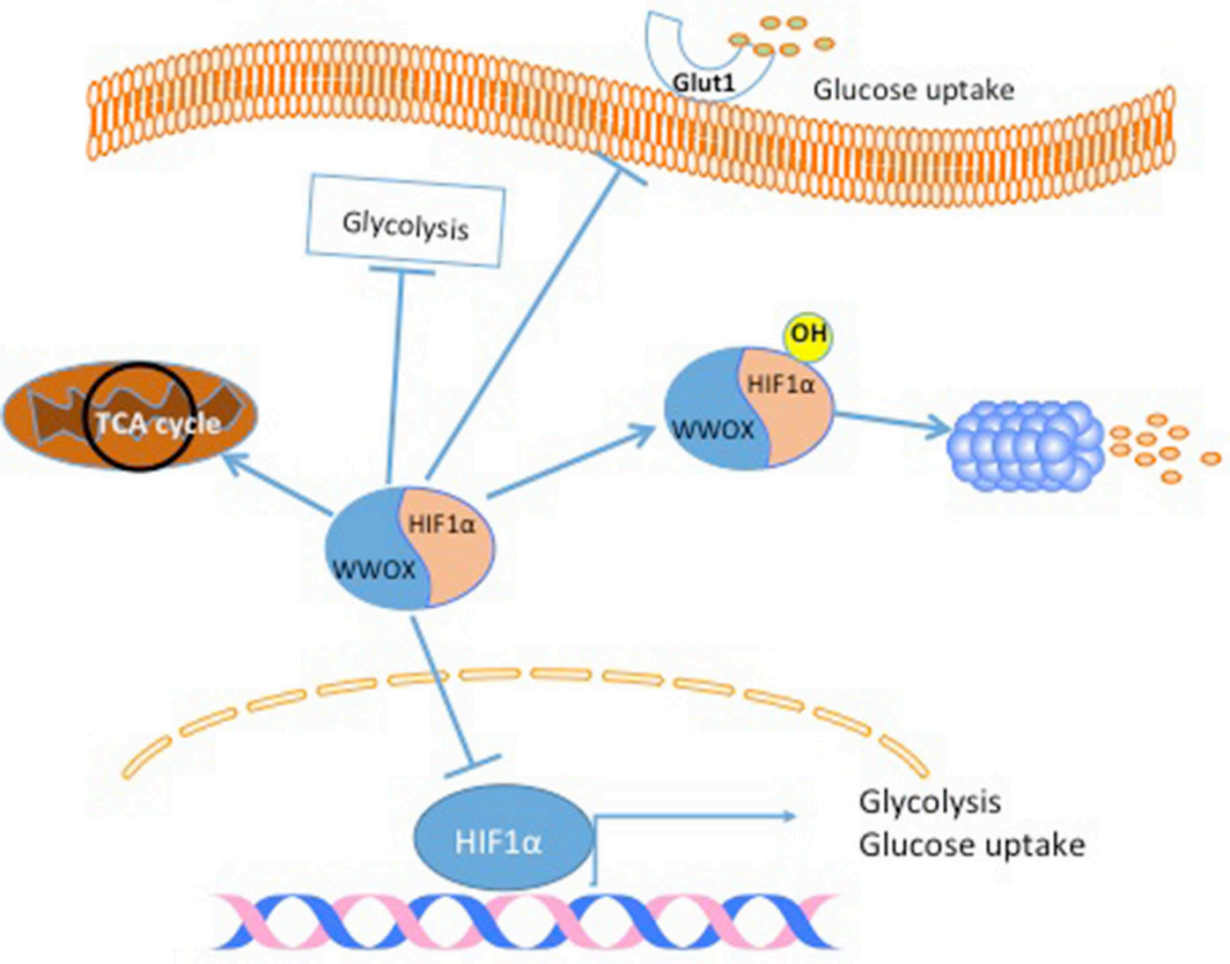
WWOX modulates cancer cell metabolism. When WWOX is present it binds to HIF1α and inhibits its transcriptional activity. Moreover, WWOX increases HIF1α hydroxylation and increase its degradation. As a consequence WWOX reduces glucose uptake, decreases glycolysis and increase mitochondrial respiration.

Another cancer hallmark connected to the tumor suppressor gene WWOX is cell metabolism. The connection between WWOX and cancer cell metabolism was established after linking WWOX protein to Hypoxia-inducible factor 1-alpha (HIF1α) function (27). In this study, it was found that WWOX interacts with HIF1α and modulates its transcriptional activity (Figure 3). In breast cancer cells, the authors showed that WWOX knock down increases HIF1α in MCF7 cells (27). Finally, they demonstrated that WWOX expression inversely correlates with HIF1α target gene Glucose transporter 1 gene (Glut1) in breast cancer tissue samples (27) (Figure 3). These findings and others all point to the fact that WWOX is very important in modulating cancer cell metabolism in general and breast cancer cells specifically. Since WWOX function is usually lost in breast cancer, it will be interesting to test whether a small molecule that resembles WWOX WW domain would inhibit the function of HIF1α and shifts cancer cell metabolism from a glycolysis dependent to a normal metabolism.

### Metastasis

Most of cancer patients die from metastasis and not from primary tumors. WWOX was also found to play a role in breast cancer metastasis. Loss of WWOX expression in breast cancer is significantly associated with the number of metastatic axillary lymph nodes and poor survival (28). In addition, WWOX was shown to play an important role in hepatocyte growth factor (HGF) mediated mesenchymal to epithelial transition (MET) in breast cancer bone metastasis (29). Another study demonstrated that the chemotherapeutic drug 5-aza-2′-deoxycytidine (decitabine), increases WWOX expression and localization to the nucleus in bone metastasis xenograft model. It was proposed that the elevation in WWOX expression is responsible for altering the HGF/Met/E.cadherin signaling axis and thus reducing breast cancer metastasis to the bone (30). In a different study that examined the role of WWOX and Transcriptional coactivator with PDZ-binding motif (TAZ) in breast metastasis to bone, WWOX and TAZ increased invasiveness of the bone metastasis-derived clone 1,833 (these cells were derived from MDA MB-231 metastasis to the bone) (31). This effect on 1,833 clone was explained by the fact that overexpression of WWOX and TAZ increases E.cadherin levels, which is an event believed to be important in MET and successful bone metastasis. Interestingly, in the same study, WWOX overexpression didn't alter E.cadherin levels and inhibited the invasion capability of the 1,833 clone parental cells, MDA MB-231 (31). These findings and others indicate that at least some of WWOX suppressive functions are cell context specific, and that experiments questioning WWOX functions should be better designed and defined.

### Questioning WWOX Suppressive Functions

Although the compelling evidence that indicates with no doubt that WWOX is a tumor suppressor gene, the presence of WWOX in a fragile locus in the genome raises suspicion about its identity of being a classical tumor suppressor gene. As well, its localization in a fragile sequence raises another question on why such an important gene lies in a damage prone locus? The answer to this question might be that loss of WWOX can act as an early alarm for the cell to respond to different types of insults that can lead to cell stress and DNA damage.

Moreover, WWOX tumor suppressive function was questioned in different research articles. For example, in a publication by Watanabe et al., it was found that WWOX is expressed in 48 cell lines out of 49. Moreover, it was shown that, using immunohistochemistry staining, WWOX is not down regulated in cancer tissue (32). However, a careful inspection of the data presented by Watanabe et al. showed the following: first, there was no normal control cells to which the expression of WWOX was compared and second, the expression of WWOX in the tested cell lines varied and there were at least 16 cell lines that showed a very low WWOX expression when compared to, for example, MCF7 cell line. These results indicate that WWOX loss in cancer is not a black and white phenomenon, but like many other tumor suppressor genes, WWOX expression is very heterogeneous in cancer samples. In addition, the expression of a tumor suppressor gene in a cancer sample or cell line does not preclude it from being a tumor suppressor gene, since a tumor suppressor gene might be expressed in a cell line but is not functional. This might be due to a posttranslational modification, mislocalization or the lack of its partner in a specific tumor type. Another paper showed that WWOX might have a role in cancer progression toward a pre metastatic state *in vivo* (33). In this article, Chang et al. showed that WWOX1 and 2 expression upregulation and Tyr33 phosphorylation correlates with the progression of breast cancer to a pre-metastatic state. Although the authors showed evidence that WWOX is overexpressed in pre-metastatic cancer, the conclusion that WWOX may play function in tumor progression might not be accurate for the following reasons. First, the authors tested the expression of WWOX on different tumor grades obtained from different patients and not on different stages from the same patient, which means that the authors didn't compare the staining pattern to the baseline of the same patient. Second, the fact that WWOX expression is induced in pre-metastatic stage of breast cancer does not exclude the possibility that WWOX expression is induced in order to suppress tumor progression rather than promoting it, analogous to p53 stabilization after stress. These controversial findings indicate that the molecular mechanisms that regulate WWOX function as well as the molecular functions mediated by WWOX should be investigated in more depth since at least WWOX partners can have dual and controversial functions. For example, ITCH, a WWOX partner and a WW-domain protein, was found to play an important role in DDR and genomic stability (anti-tumorigenic effect) (23, 24). On the other hand ITCH in the context of the Hippo pathway was shown to have a pro-tumorigenic function (34, 35). This raises the question; How does WWOX affect ITCH pro-tumorigenic function? Does it antagonize ITCH function and suppresses tumor growth? Which is the expected scenario, or does it work synergistically with ITCH like under DDR and thus promotes tumorigenesis?

A very interesting aspect of WWOX tumor suppressive function is changing the signaling pathway of different proteins to achieve the same outcome. For example, p73 is a tumor suppressor gene that induces apoptosis through its transcriptional transactivation function. When WWOX interacts with p73, paradoxically after WWOX activation with the Src oncogene, it sequesters p73 in the cytoplasm and inhibits its transactivation function. Although this has a negative effect on p73 nuclear function, it enhances p73-WWOX interaction in the cytoplasm leading to apoptosis. This raises the following questions; why does WWOX interfere with p73 nuclear pro-apoptotic function to induce apoptosis via a different mechanism? Is cell context the only reason for this? Or is it because WWOX's pro-apoptotic function is superior to p73 pro-apoptotic function?

Although the controversy about WWOX function, it will be exciting to try to test how would peptides that resemble WWOX WW domains affect cancer cell behavior to evaluate their therapeutic functions.

## Hippo Pathway and Breast Cancer

**Figure 4:**
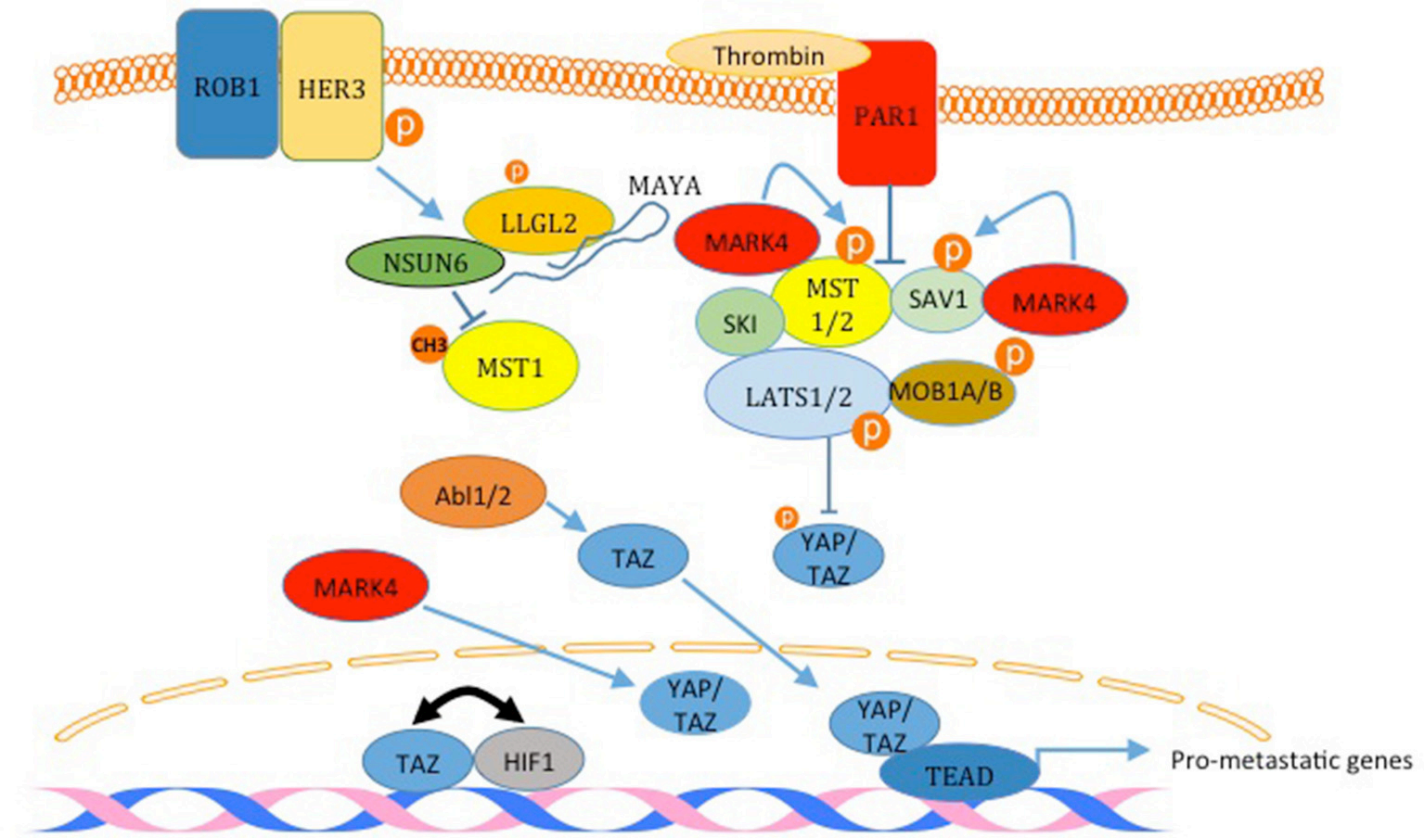
Inactivation of the Hippo pathway induces breast cancer metastasis. The factors labeled in red are examples of proteins that inactivates the Hippo pathway and induces metastasis. PAR1 activation and the kinase MARK4 destabilize the core complex and inhibits its activity. MARK4 can increase nuclear YAP and TAZ too. The activation of HER3 by ROB1 leads to the assembly of a complex between MAYA ncLRNA, LLGL2 and the methyl transferase NSUN6 that methylates and inactivates MST1. Abl kinases can also increase nuclear TAZ. Finally, TAZ and HIF1 and reciprocally act as transcription co activators for each other. The only Hippo pathway activator shown here is SKI which binds LATS2 and MST1 and strengthen the core complex.

The Hippo pathway is a highly conserved pathway that regulates many cellular functions including cell proliferation, growth, differentiation and apoptosis (2). The pathway is composed of mainly a core cascade of kinases that include Macrophage-stimulating protein 1/2 (MST1/2) and Large tumor suppressor kinase 1/2 (LATS1/2). These kinases, when activated lead to the phosphorylation, sequestration and inactivation of two WW domain downstream effector proteins; yes-associated protein (YAP) and TAZ (Figure 4). The pathway is enriched with WW domain mediated interactions on the level of upstream modulators, core components, as well as downstream effectors (Figure 4) (1, 36, 37).The pathway is either inactivated or harbors a mutation in one of its components in many types of cancer; including breast cancer.

### Animal Models and Clinical Data as Evidence for YAP/TAZ Roles in Breast Cancer

In different animal models, YAP and TAZ functions supported tumor initiation and progression (38, 39). In the clinical context, TAZ was shown to have a negative prognostic effect that correlates with shorter disease-free survival (DFS) of breast cancer patients (40) and negatively correlates with treatment outcome (41). In luminal A breast cancer samples, YAP1 expression levels negatively correlates with Estrogen positive (ER+) samples, and positively correlates with proliferation in ER- samples. Moreover, in the same study, low YAP1 levels correlated with impaired response to tamoxifen treatment (42). In a different study YAP nuclear levels were higher in metaplastic breast cancer tumors when compared to triple negative tumors (43). In another study that examined the expression of YAP in different molecular types of breast cancer, cytoplasmic and phospho-YAP levels were elevated in HER-2 breast cancer type. In this molecular subtype, cytoplasmic YAP expression concurred with poorer disease free survival. In addition, nuclear YAP staining was associated with shorter overall survival (44). In contrast to all findings that revealed that YAP expression correlates with a more aggressive breast cancer and with a poor survival and disease free rates, a recent study by Cao et al. demonstrated that YAP expression is associated with the low grade type of breast cancer luminal A. In addition, the study revealed that YAP expression correlates with favorable DFS and overall survival in patients with luminal A breast cancer and with favorable DFS association among patients with invasive ductal carcinoma, luminal B (HER2-), and luminal B (HER2+) breast cancers (45). These results indicate that there is still some controversy about YAP expression and its correlation with clinical outcome in breast cancer clinical samples. Of course these controversies can be related to different reasons starting from very technical issues related the staining techniques, the antibodies used in each study to data scoring and data analysis. While these can still be possibilities, other biological reasons can still be an explanation. For example, other genetic factors and expression profiles of many other different proteins, in addition to YAP status, that were not tested in most of the studies can provide explanations.

### YAP/TAZ Roles in Breast Cancer Cell Transformation and Tumorigenesis

In addition clinical data, it is obvious that YAP and TAZ play critical roles in breast tumorigenesis. For example, YAP over expression in MCF10A, non-transformed mammary gland cells, increases cell proliferation, anchorage independent growth and inhibits apoptosis (38). On the other hand, YAP knockdown results in cell growth inhibition *in vitro* and *ex vivo* tumor formation (46). In different studies, YAP oncogenic function was related to Hippo pathway malfunction. For example, it was demonstrated that LATS1degradation mediated by ITCH releases YAP function and enhances breast cell tumorigenicity *in vivo* and *in vitro* (34, 35). Similar to YAP, TAZ was shown to be involved in breast tumorigenesis. While overexpression of TAZ in MCF10A cells causes transformation morphologic changes, TAZ knockdown reduces their tumorigenicity *in vitro* and *in vivo* (47). Similar to YAP, uncoupling of TAZ from the inhibitory effect of the Hippo pathway, upon LATS1 knock down, enhances TAZ-mediated phenotypes (48). Moreover, TAZ transforming activity was shown to be dependent on Transcriptional enhancer factor TEF-1 (TEAD) transcriptional activity (49). On the mechanism level, it was demonstrated that coordination between YAP, TAZ and Transforming growth factor beta (TGFβ executes a specific pro-tumorigenic transcriptional program that is important for overcoming the anti-tumorigenic effect of TGFβ. In addition, TAZ was shown to stabilize Krüppel-like factor 5 (KLF5) which promotes breast cell proliferation and tumorigenesis by competing with the E3 ubiquitin ligase WW-domain protein1(WWP1) on KLF5 (48).

### YAP/TAZ Roles in Breast Cancer Metastasis

The link between the Hippo pathway and YAP/TAZ mediated breast cancer metastasis was proved in different publications. For example, Ski interacts with LATS2, Sav, Mob, and Mer and facilitates the phosphorylation of YAP and TAZ, which leads to inhibition of YAP/TAZ induced transformation and epithelial to mesenchymal transition (EMT) (Figure 4) (50). In another study, activation of human epidermal growth factor receptor 3 (HER3) by ROR1 was shown to recruits the adaptor protein LLGL2, lncRNA MAYA (MST1/2-Antagonizing for YAP Activation), and methyltransferase NSUN6 (Figure 4). This leads to methylation and inactivation of MST1, which liberates YAP and increases breast cancer cell metastasis to the bone (51). Recently, it was demonstrated that TWIST, via the activation of thrombin receptor PAR1, inactivates the Hippo pathway and induces EMT and promotes breast cancer cell invasion (Figure 4) (52). Also, it was shown that TAZ and HIF1α interaction progresses breast cancer metastasis (Figure 4) (53, 54). Different kinases were also shown to promote breast cancer cell migration through inactivating the Hippo pathway. These kinases include ABL and MARK4 kinases (55, 56). In addition to these studies, different other studies have shown that YAP and TAZ lead to breast cancer migration, invasion and metastasis (38, 40, 47, 57, 58).

### Hippo Pathway and BC Stem Cells

**Figure 5:**
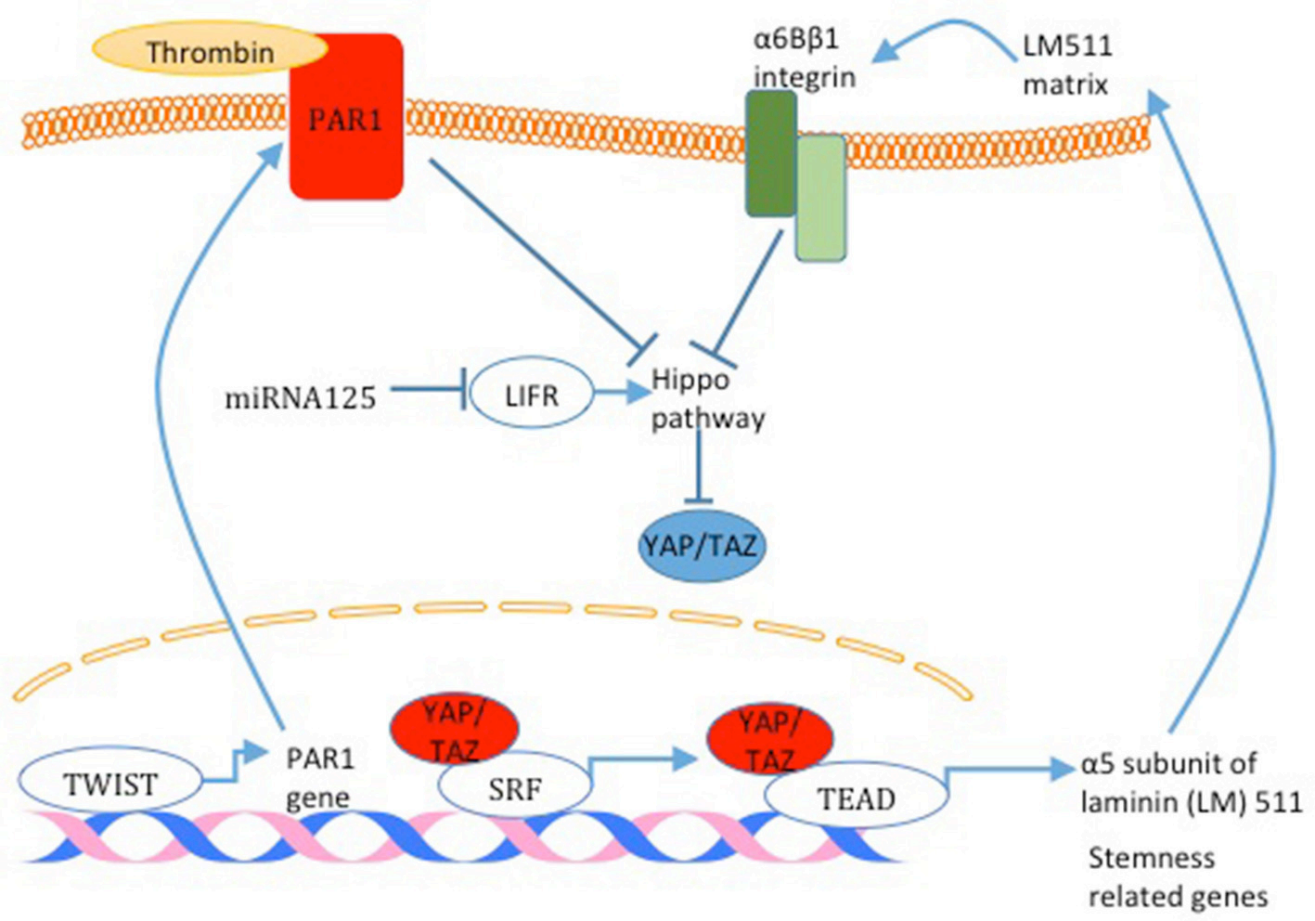
Hippo pathway activity and breast cancer cell stemness. Different mechanisms responsible for Hippo pathway inactivation are illustrated in this figure. miRNA125 targets the Hippo pathway activator LIFR and induces TAZ activity. Also, TAZ was found to activate the expression of α5 subunit of laminin (LM) 511 and the formation of a LM511 matrix. Reciprocally, LM511 activates α6Bβ1 integrin that activated TAZ. Finally, TWIST increases PAR1 expression which consequently inhibits the Hippo pathway and induces TAZ function. TAZ activation through all these mechanisms increases the expression of breast cancer cell stemness related genes.

The link between BCSC and the Hippo pathway was initially established in a study that demonstrated that TAZ is required to sustain self-renewal and tumor-initiation capacities in BCSCs (59). TAZ was also shown to be overexpressed in tumors derived from BCSC compared to tumors derived from non-BCSC (40). In addition, overexpression of TAZ in non-transformed basal cells (MCF10A) confers cancer stem cell phenotype in these cells (60). Moreover, TAZ is indirectly tied to breast cancer stemness after the finding that revealed that miRNA125, by targeting the Hippo pathway and activator leukemia inhibitory factor receptor (LIFR), induces TAZ activity and breast cancer stemness (Figure 5) (61). On the mechanism level, TAZ was found to be involved in a positive feedback loop that activates the expression of α5 subunit of laminin (LM) 511 and the formation of a LM511 matrix. Reciprocally, LM511 activates α6Bβ1 integrin, which activates TAZ and promotes breast cancer stemness (Figure 5) (62). Moreover, TWIST increases PAR1, which was shown to inhibit the Hippo pathway and induce breast cancer stemness phenotype by activating TAZ (Figure 5) (52). YAP was also connected to breast cancer cell stemness. YAP overexpression upregulates mammary stem cell (MaSC) signature genes (63) (Figure 5). Moreover, it was concluded that YAP is more important in cancer stemness in basal-like breast cancer compared with luminal-type breast cancer and that YAP correlates with poor relapse-free survival specifically in basal-like breast cancer compared to luminal-type breast cancer (63).

Recently, in breast cancer patient samples, high YAP expression profiles correlated with EMT and stemness gene signature enrichment (64). Finally, in an attempt to study the role of Hippo pathway in mammary gland development and tumor formation using gland reconstitution, it was found that Hippo signaling is involved in mammary tumor formation but not essential for mammary gland development (65).

### Immune Evasion

Immune evasion, or escape from immune system control is one of Cancer “Hallmarks” (21). Paradoxically, it is documented that the presence of intact and functional immune system can prevent or promote carcinogenesis by a process known as “Cancer Immuno editing” (66–68). Accordingly, immunotherapy gained interest and is seen promising in curing cancer. Recently, it has been published that either deletion of LATS1/2 or overexpression of YAP/TAZ increases tumor immunogenecity and thus enhance its destruction by the immune system *in vivo* (69). These findings, to some extent, contradict previous work that showed that LATS1/2 are tumor suppressor genes and YAP/TAZ are oncogenes. In contrast to these findings about the immune activating function of YAP, it was shown that YAP overexpression induces an immunosuppressive environment (70, 71). In breast cancer, a recent work revealed that while LATS1/2 and MST1/2 reduce the expression of the immune checkpoint molecule PD-L1 in cancer cells, YAP/TAZ have an opposite effect (72). Since PD-L1 has an immunosuppressive function, these results are in concordance with the previous results that YAP/TAZ are immunosuppressive (70, 71) rather than being more immunogenic (69). Since PD/PD-L1 interaction is currently targeted for cancer immunotherapy, these findings suggest that YAP/TAZ might be used as a predictive factors for PD/PD-L1 based cancer immunotherapy.

### Therapeutic Potential of the Hippo Pathway

Based on various roles played by the Hippo pathway and its downstream effectors YAP and TAZ in breast cancer, it seems that modulating the activity of the Hippo pathway holds a hope of being therapeutic target in breast cancer. In fact there are different compounds that were used and showed an effect on YAP/TAZ functions. For example, Verteporfin treatment inhibits proliferation, invasion and migration of the breast cancer cell line MDA MB231 (73). This effect of Verteporfin was observed with other types of cancer (74–77). Other molecules that have anti-tumorigenic effect by attenuating YAP/TAZ function are statins, which inhibit the synthesis of geranylgeranyl pyrophosphate produced by the mevalonate cascade. This product stimulates Rho GTPases and in turn activates YAP/TAZ by inhibiting their phosphorylation. Thus, by inhibiting mevalonate cascade, statins inhibit YAP/TAZ activity (78). In breast cancer, simvastatin was shown to down regulate the expression of the downstream gene RHAMM. This attenuates breast cancer cell invasion and motility induced by signal–regulated kinase (ERK). Of note, these outcomes were revealed to be independent of MST and LATS kinase activities (79). In another study, combinations of Dasatinib and statins (which induce YAP/TAZ phosphorylation) and pazopanib (which induces proteasomal degradation of YAP/TAZ), with other anti-cancer drugs, like doxorubicin and paclitaxel, inhibited YAP/TAZ-dependent breast cancer cell proliferation. For example, in MDA MB231 cell line, which is YAP/TAZ dependent, these combinations synergistically reduced cell viability and tumorigenicity *in vitro*. On the other hand, in MCF7 cells, which are YAP/TAZ independent, these combinations didn't show the effects observed in MDA MB231 (80). The fact that different cells respond differently to YAP/TAZ inhibition indicates that other biomarkers play a role in cell response to therapy and not only the presence or absence of a single molecule like YAP and TAZ. Thus, future studies should address the effect of targeting YAP/TAZ, and concentrate on wider analysis and profiles of other proteins related to the functions of YAP/TAZ. Also, as discussed below, there is no consensus about YAP functions in breast carcinogenesis. In addition, it is always mandatory to perform drug screening experiments in a model that recapitulates what happens in tumors *in vivo*. In relation to this, while different studies revealed that Hippo pathway activation or YAP/TAZ inactivation results in an anti-tumorigenic outcome, It was found that inactivation of the Hippo pathway (by LATS1/2 knockdown or YAP/TAZ overexpression) increases tumor immunogenecity and thus enhances its destruction by the immune system *in vivo* (69). Over all, although the results from Hippo pathway manipulation in cell lines and animal models makes it a potential therapeutic target in cancer, it seems that a lot is still needed to be done to prove the reproducibility of these results in breast cancer patients.

### Controversies About YAP Oncogenic Functions

As discussed above, compelling evidence proved that TAZ has only pro-tumorigenic functions. On the other hand, research regarding YAP function in cancer still holds some controversies. For example, YAP interacts with p73 and increases its transcriptional activity (81). This YAP-p73 interaction is important in driving p73 gene-target specificity in response to DNA damage (82). Moreover, under DNA damage YAP1 protects p73 from ITCH mediated degradation and thus induces p73-dependent apoptotic response (83). In breast cancer too, YAP has tumor suppressive functions. For example, it was demonstrated that YAP locus undergoes loss of heterozygosity, which might indicate that the YAP locus (11q22–23) harbors a tumor suppressor (84, 85). In addition, it was demonstrated that YAP has a pro-apoptotic function that can be inhibited by AKT oncogene (86). Moreover, knockdown of YAP in breast cancer cell lines suppressed anoikis, increased migration and invasiveness, inhibited response to taxol and enhanced tumor growth in nude mice (87). Finally, it was revealed that MicroRNA-200a promotes anoikis resistance and metastasis by targeting YAP1 in human breast cancer (88).

This controversy about YAP tumor suppressor and oncogenic functions can be explained, at least in part, by the fact that YAP has different isoforms which are differentially expressed in tissues as a result of differential splicing (89). These isoforms, as one might speculate, have different functions under different cell physiological contexts or between different cell types. In fact, most of the publications that studied YAP function in cancer did not acknowledge which YAP isoform was used or investigated, which makes it difficult to compare findings obtained from these different studies. For example, overexpression of hYAP1-2γ in mammary gland cells promotes oncogenic phenotypes including protection from apoptosis (89). On the other hand, overexpression of hYAP1-2α in squamous carcinoma cells induced apoptosis (90). Moreover, in a recent paper that studied the transcriptional potencies of YAP isoforms, it was shown that splice variant insertions in the C-terminus, which lead to the disruption of YAP leucine zipper, decreased YAP transcriptional activity (91). In conclusion, the discrepancy about YAP function in breast cancer and the presence of different YAP isoforms that seem to have different effects on the Hippo pathway signaling, ensures the need for better understanding of the role of the different YAP isoforms in breast cancer. This will help in the development of better YAP/TAZ based potential breast cancer treatments.

## E3 Ubiquitin Ligases and Breast Cancer

Ubiquitination is an important post translation modification that alters protein function, either by destabilizing it, or through changing its subcellular localization. The fate of the ubiquitinated protein seems to depend on the length and architecture of the ubiquitin chain. For example, while K48-linked polyubiquitin chains mediate protein degradation in the proteasome, K63-linked polyubiquitin chains change protein subcellular localization under different cell physiological situations (92). E3s can be generally classified into three subfamilies: (1) The homologous to E6-AP carboxyl terminus (HECT) domain-containing E3s; (2) Finger domain-containing E3s and; (3) and the U box E3s (2). Ubiquitination involves a cascade of reactions catalyzed by 3 different enzymes; E1 (ubiquitin activating), E2 (ubiquitin conjugating) and E3 (ubiquitin ligating) enzymes. The specificity of this reaction is determined by the E3 ligase, which binds in a specific manner to a specific substrate. Ubiquitination is involved in many biological processes including DNA damage response, cell proliferation, apoptosis, cell cycle, transcription, and immune response (93–96). Deteriorations in the ubiquitination system are connected to the development of different diseases including; autoimmunity, and inflammatory diseases (97), neurodegeneration (98) cardiac diseases (99), and cancer (1, 2, 93, 100). In cancer, the ubiquitin system regulates different cellular processes and targets involved in carcinogenesis including cell cycle, p53, transcription, DNA repair, cell signaling and apoptosis.

NEDD4 or NEDD4-like E3s interacts with their target proteins via their WW domains. These E3s are involved in different processes in different types of cancer including breast cancer as discussed below.

### E3s Role in Breast Cancer Growth and Survival

**Figure 6:**
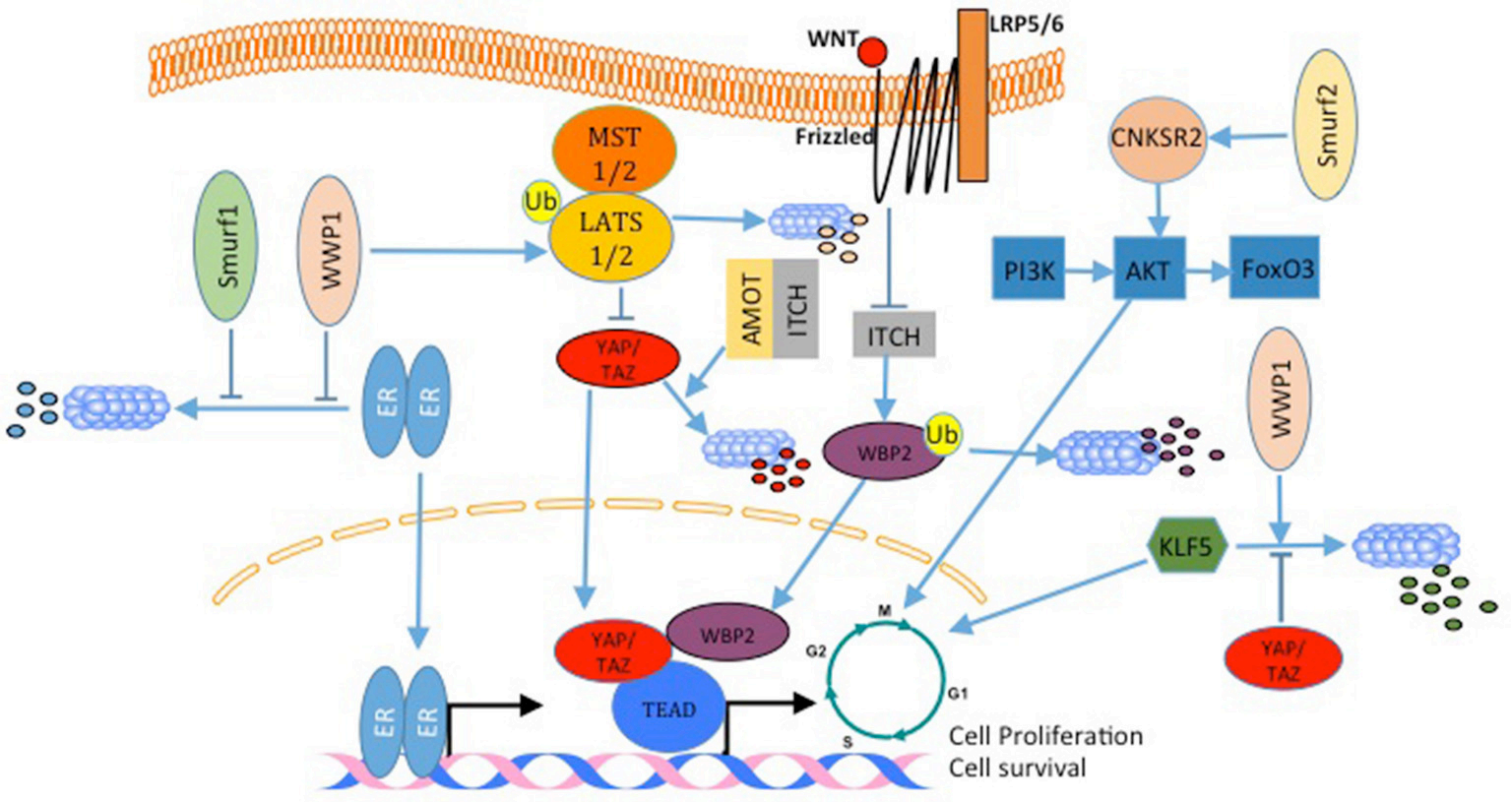
E3 ligases regulate breast cancer cell proliferation. In breast cancer, both, WWP1 and SMURF1 stabilize ER and enhances its activation. WWP1 can also ubiquitinate and degrade LATS1 and liberates YAP/TAZ transcriptional activities. In contrast to this WWP1 can also target the pro-proliferative factor KLF5 and inhibit cell growth. This WWP1 activity can be inhibited by YAP and TAZ. SMURF2 supports cell proliferation by activating the PI3K-A-AKT-FoxO3 pathway. ITCH with angiomotin lead to YAP degradation and inhibits YAP proliferative activities. Wnt signaling inhibits WBP2 degradation by ITCH, and increases its transcription transactivation function. All these are mechanisms that activate cell cycle and transcription of pro-proliferative genes.

Estrogen receptor alpha (ERα) is expressed in almost 70% of breast cancers and promotes estrogen-dependent cancer cell proliferation and tumor progression. Recently, it was shown that Secondary Metabolite Unique Regions Finder 1 (SMURF1), via its HECT domain, interacts with and stabilizes ER, and that depletion of SMURF1 decreases ERα-positive cell proliferation *in vitro* and *in vivo* (101). This pro-proliferative and pro-oncogenic effect of SMURF1 in breast cancer was found to be the case with SMURF2 too (Figure 6). SMURF2 silencing in human breast cancer cells results in a low tumorigenicity of the cells *in vitro*, and also, arrests cells in G0/G1 phase of cell cycle (102). Analysis of the mechanism that results in these phenotypes revealed that SMURF knockdown destabilizes CNKSR2 (connector Enhancer Of Kinase Suppressor Of Ras 2) protein in the cell. Of note, this effect is mediated by WW domain protein-protein interaction (102). Another E3 that was connected to breast cancer cell proliferation is WWP1. WWP1 enhances cell proliferation after polyubiquitination and proteosomal degradation of LATS1 (Figure 6) (103, 104). In this context, while over-expression of WWP1 enhances cell proliferation in LATS1-positive MCF10A mammary epithelial cells, knockdown of WWP1 in MCF7 breast cancer cells reduces their proliferation (103, 104). Also, WWP1 supports cell proliferation and survival by targeting ErbB4 for proteosomal degradation (105, 106). This pro-proliferative effect of WWP1 on breast cancer is in concordance with other results that showed that WWP1 stabilizes ER (Figure 6), which supports cell proliferation in ER+ cells. When WWP1 is knocked down, cells become more sensitive to tamoxifen treatment (103). While all the studies discussed above point out to the fact that WWP1 is a pro-proliferative factor, two independent studies published by the same group showed that WWP1 might have an anti-proliferative role through targeting KLF5 (Figure 6), which is a transcription factor that promotes breast cell proliferation and survival (107, 108). In these studies, both YAP (107) and TAZ (48) prevented the E3 ubiquitin ligase WWP1 from ubiquitinating and sending KLF5 for degradation (Figure 6). Recently, Lim et al. demonstrated that ITCH overexpression reverses breast cancer progression mediated by Wnt signaling. The authors showed that wnt signaling blocks ITCH mediated degradation of YAP/TAZ transcriptional coactivator WBP2 (Figure 6), and thus promotes breast cancer cell proliferation (109). On the same principle, but utilizing a different mechanism, in a different cellular context, Amot130 and ITCH were shown to promote the ubiquitination, degradation and inhibition of YAP function in response to serum starvation (110) (Figure 6).

### E3s Modulate Breast Cancer Cell Migration, Invasion, and Metastasis

**Figure 7:**
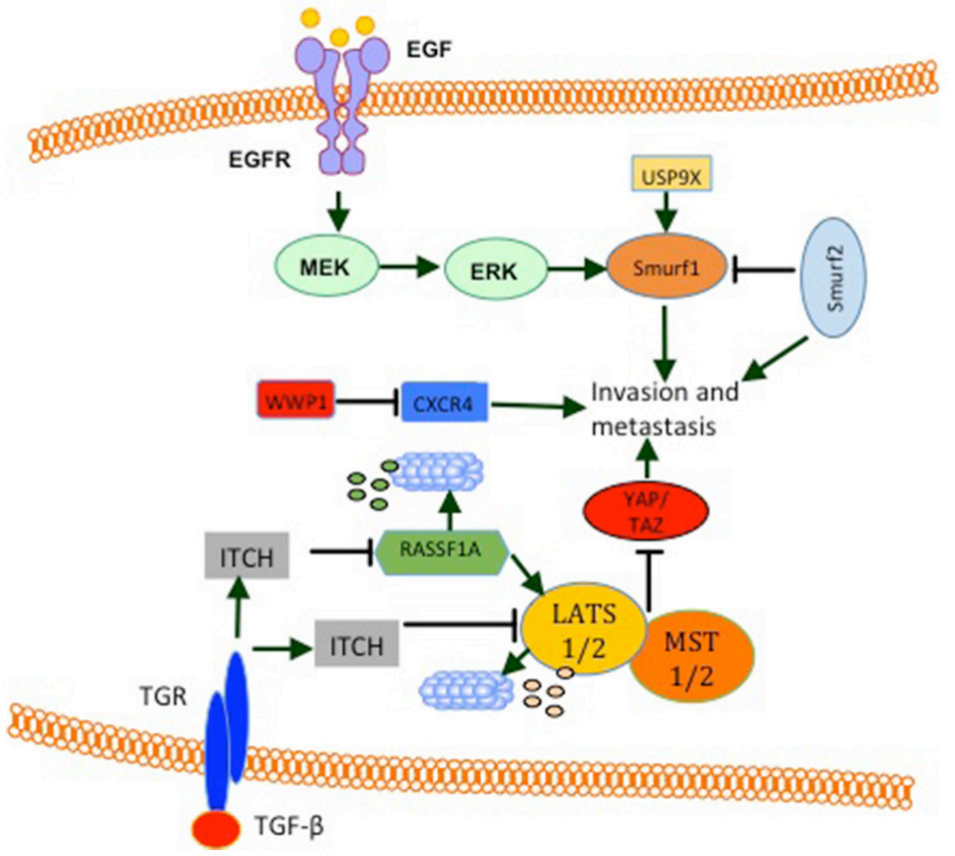
E3 modulate breast cancer cell invasion and metastasis. EGF induces the activity of ERK signaling pathway that increases SMURF1 pro-invasive activities. SMURF1 is also stabilized by USP9X. SMURF2 has both pro- and anti- invasive and metastatic functions. By inhibiting SMURF1, SMURF2 inhibits cell invasion and metastasis. On the other hand SMURF2 was shown to induce cell invasion and metastasis. WWP1 reduces bone metastasis by inhibiting CXCR4. ITCH induction by TGF-β or other unknown mechanisms promotes cell invasion and metastasis by sending RASSF1 and LATS1 (Hippo pathway activators) for proteosomal degradation.

WW domain-containing E3s function in tumor metastasis was also established. Analysis of the expression level of SMURF2 protein revealed that it is elevated in 30% of mammary ductal carcinomas as well as in aggressive and metastatic breast cancer cell line MDA MB231 (111). In this article, it was disclosed that, while SMURF2 knockdown lowers aggressiveness and motility of breast cancer cells, its overexpression promotes metastasis *in vivo* and *in vitro* (111). These findings are supported by results obtained by David et al. (102), who showed a high expression level of SMURF2 in the aggressive MDA-MB-231 cell line compared to other less aggressive cancer cell lines. Moreover, they showed that SMURF2 silencing in human breast cancer cells decreases cell migration/invasion *in vitro* (102). Also, in tissue samples, SMURF2 protein levels were high in infiltrating ductal carcinoma when compared to normal tissue. These findings support the notion that SMURF2 supports invasiveness and metastasis in breast cancer. In fact these findings are in controversy with a publication by Liu (112), which demonstrated that SMURF2 expression is downregulated in triple negative metastatic tumors in comparison with either benign lesions or ductal carcinoma *in situ*. Also, human triple-negative breast cancer cell lines such as BT549, MDA-MB-436, DU-4475 and MDA-MB-468 cells showed significantly lower expression of SMURF2 protein, compared to ER + or HER2+ cell lines (112), indicating that SMURF2 might play an inhibitory function in the context of breast cancer invasion and metastasis. Indeed, two other studies supported this notion. in these studies, SMURF2 knockdown was shown to lead to a more aggressive and more migratory metastatic phenotypes *in vitro* and *in vivo* (113, 114). Interestingly, in one of these studies, SMURF2 inhibited cell invasiveness by reducing SMURF1 levels (113). In line with these finding, SMURF1 was shown to be important for EGF mediated cell migration and invasion (114). In addition, SMURF1 expression is elevated and required for MDA-MB-231 breast cancer cells motility. In these cells, Ubiquitin Specific Peptidase 9 X-Linked (USP9X) stabilizes endogenous SMURF1, and depletion of USP9X leads to down-regulation of SMURF1 and significantly impaired cellular migration (Figure 7) (115). Another WW domain containing E3 ligase that was shown to play a role in breast cancer migration and metastasis is WWP1. WWP1 knockdown in MDA-MB-231 breast cancer cells results in more osteolytic lesions and increases tumor area in bone marrow of mice when injected into the left ventricle of the heart (116). In this study, WWP1 knockdown reduced CXCL12-induced CXCR4 lysosomal trafficking and degradation (Figure 7) (116). These results proved that WWP1 inhibits breast cancer cell metastasis to the bone. ITCH E3 ligase was also connected to tumor invasion and metastasis. It was shown that in order to mediate TGF-β-induced breast cancer invasion, ITCH must degrade Ras association domain family 1 isoform A (RASSF1A) (Figure 7). As a consequence, Hippo pathway effector YAP1 associates with SMADs and results in their nuclear translocation (117). The pro-tumorigenic activities of ITCH were shown to be mediated also through LATS1. ITCH was shown to interact with LAST1 and send it to proteosomal degradation. As a consequence, LATS1 degradation mediates YAP translocation into the nucleus and induces its transcriptional activities. This YAP activation induces EMT and breast cancer invasion and metastasis *in vitro* and *in vivo* (34, 35).

### Apoptosis

Previous studies that investigated WWP1 expression level in breast cancer revealed that WWP1 knockdown significantly induces cell growth arrest and apoptosis in breast cancer cell lines by activating different caspases. In addition, it was found that overexpression of WWP1 in immortalized breast epithelial cell lines MCF10A and 184B5 promotes cell proliferation (118). In concordance with these findings, it was found that WWP1 depletion activates the TNF-related apoptosis-inducing ligand (TRAIL) extrinsic apoptotic pathway (Figure 1). Moreover, a correlation was found between the expression levels of WWP1 in four breast cancer cell lines and TRAIL resistance, but not tumor necrosis factor alpha (TNFα) and doxorubicin resistance (108). Another E3 ligase that plays a role in apoptosis is ITCH, which interacts with and destabilizes the tumor suppressor gene Ras association domain family Member 5 (RASSF5). This interaction inhibits RASSF5-mediated G1 phase transition of cell cycle as well as apoptosis (119) (Figure 1).

### E3s and Genomic Stability

SMURF2^−/−^ aging mice develop different types of tumors including breast cancer. In an attempt to explain this phenotype, it was discovered that SMURF2 deletion leads to the stabilization of the RNF20/hBre1A (Figure 2), the major ubH2B-specific E3. This upregulation of RNF20 leads to changes in chromatin landscape and genomic instability (120). Furthermore, the same group elucidated that SMURF2, by stabilizing Topoisomerase IIa (Topo IIa) (Figure 2), protects cells from DNA damage and genomic instability. They also found that SMURF2-depletion leads to reduced cell ability to resolve DNA catenanes and to pathological chromatin bridges formed during mitosis, which are traits that were observed in Topo II deficient cells and are also a hallmark of chromosome instability (121). ITCH was also connected to genomic stability. In response to DNA damage, ITCH activity leads to nuclear accumulation of WWOX through its K63-linked ubiquitination at lysine residue 274. Nuclear WWOX then interacts with ATM or ATR (under different types of insults) and enhances their activation and thus enhances cellular DNA damage response and genomic stability (Figure 2) (23, 24).

### E3s and Breast Cancer Therapy

As described above, E3s are involved in many cancer related processes. Thus, they might be good targets for cancer treatment. However, some facts might limit the potential of E3s to be targets for cancer therapy. For instance, E3s can play antagonistic effects under different cellular contexts. For example, ITCH was shown to have both, anti-tumorigenic as well as pro-tumorigenic activities by targeting different proteins. More than that, some E3s can target related proteins that have antagonistic functions. For example p63 is a WWP1 target. p63 has different isoforms that are differentially expressed in different tissues and can have even opposite functions. For example, while TAp63 is believed to sensitize cells to apoptosis, DNp63 has an opposite function. It was found that WWP1 ubiquitinates, and destructs both DNp63α and TAp63alpha. While knockdown of WWP1 increases the DeltaNp63α levels in the MCF10A and 184B5 immortalized breast epithelial cell lines and conferred resistance to doxorubicin-induced apoptosis, knockdown of WWP1 increases TAp63α level, induces apoptosis, and increases sensitivity to doxorubicin and cisplatin in the HCT116 colon cancer cell line (122). In addition to these limitations, it is well documented that a specific E3 ligase can target different substrates, which to a certain extent reduces the approach specificity and may predict the presence of side effects for E3s based therapies. Moreover, different types of ubiquitinations can result in different effects. For example while K48 type of ubiquitination is usually connected to protein degradation, K63 usually changes protein function and localization. The way the type of the ubiquitination is being selected under different cellular contexts is another issue that might impede the development of E3s based therapies. Another issue that needs to be addressed when trying to look for therapeutic targets related to ubiquitination is the fact that different E3s can have different isoforms. In breast cancer cell line T47D, six isoforms of WWP1 have been identified (123). These isoforms were shown to have different domain structures. Some of these isoforms contain or lack an N-terminal C2 domain. The distribution of these isoforms is tissue specific (123). Consequently, Flasza et al. raised the possibility that alternative forms are targeted to different locations in the cell, and thus may possibly regulate target protein selection. This means that an E3 might have different targets in different tissues or under different cellular contexts.

Chemo resistance is one important mechanism that impedes successful cancer treatment. E3s were also tied to chemo resistance (124). For example, WWP1 was shown to inhibit TRAIL induced apoptosis in breast cancer cell lines (108). Moreover, WWP1 was shown to stabilize p53 and leads to its exportation to the cytoplasm and thus inhibiting its transcriptional activity (108). These findings suggest that WWP1manipulation in p53 wild type breast cancer tumors can sensitize them to anti-cancer treatment (125).

Another example on the link between E3s and response to therapy is ITCH. In a recent publication, ITCH was shown to sensitize ER+ breast cancer cells, who acquired resistance to endocrine treatment (126). In this study, the transition from an endocrine therapy sensitive to a resistant state was accompanied with c-Jun N-terminal kinases (JNK) activation. This JNK activation seemingly resulted in ITCH phosphorylation and activation and thus c-FLIP degradation (126). In another study, in an attempt to characterize the mechanism of action of the neuregulin-non-competitive anti-HER3 therapeutic antibody 9F7-F11 that blocks the PI3K/AKT pathway and induces cell cycle arrest and apoptosis of breast cancer, it was found that 9F7-F11 activates JNK and consequently ITCH. This ITCH activation was shown to induce rapid HER3 down-regulation (127). Also, several ITCH inhibitors were identified in high throughput screening for putative ITCH modulators. One of these identified ITCH inhibitors was clomipramine—a clinically useful antidepressant drug. Treatment of different cancer cell lines including breast cancer cell lines with clomipramine, or its homologs, reduced cancer cell growth, and synergized cancer cell killing by gemcitabine or mitomycin by blocking autophagy (128). In conclusion, these findings and others indicate that E3s themselves can be targets for cancer therapies, or can be biomarkers for the prediction of a specific therapy response.

## Concluding Remarks and Future Directions

In conclusion, studies reviewed here demonstrate that WW domain-containing proteins are critical players in breast cancer initiation and progression. As discussed above, WW domain-containing proteins can act as singles, or involved in the assembly of signaling complexes like in the case of the Hippo pathway. They can act as oncogenes or tumor suppressors by altering different cancer related processes. However, some of WW domain-containing proteins could act as either tumor suppressors or oncogenes with some controversies about the identity of some of these proteins.

As we discussed above, these controversies about the functions of some of WW domain-containing proteins could be related not only to cell context, but also to their interactions and their partners. Moreover, we explained that these controversies could stem from the presence of different isoforms of the same protein. These isoforms can have different cell and tissue distribution patterns and thus may have different partners and targets. Thus, in future research, it might be very important and necessary to mention or identify the isoform studied in a specific context. For example the current literature does not specifically mention which YAP or WWP1 isoform(s) are addressed in a specific study, which makes it difficult compare and judge controversial results obtained. These controversies and different functions of these proteins can be resolved also by future studies that will be based on more global approaches that will study the proteome or even the interactomes of these proteins in different cell contexts and in relation to different cancer related processes regulated by WW domain-containing proteins. Such studies, in addition to illustrating the mechanism(s) of action of the different WW domain-containing proteins, they will identify other biomarkers involved in the execution of phenotypes related to the different WW domain-containing proteins. In fact the identification of such biomarkers and elucidation of exact mechanism of action can be also beneficial in the prediction of therapeutic outcomes related to the manipulation of WW domain-containing proteins in the future.

Another future direction that should be followed in the context of WW domain-containing proteins and breast cancer is the identification of new such proteins. It is predicted that there are many other different WW domain-containing proteins that will have impact on breast tumorigenesis. The fishing for such proteins can be based on pulling down WW proteins using known PY rich domains, or by developing and using different softwares to help predict the presence of WW proteins in the human genome.

The use of WW domain-containing proteins as drug targets is still a premature idea that needs intensive future research. In this regard, one can raise the question of what to target in these proteins? Shall a drug target the catalytic activity of these proteins? Or their interactions by targeting their WW domains? Or target even their partners and targets? The answer to these questions will never be easy with our current knowledge about these proteins. However, we can speculate about the complexity in choosing which proposed strategy would be a successful one. For example, upon targeting the catalytic domain of different WW domain-containing proteins, a drug can target different proteins that have antagonistic functions in the context of cancer. For instance, if drugs were developed to target the evolutionary-conserved catalytic HECT domain, these drugs will target for example SMURF1 and SMURF2, which were shown to have antagonistic functions in breast carcinogenesis, and this, theoretically, might make such a strategy a failing one. In choosing to target WW domains in order to disrupt protein-protein interaction is also complex. Although there are different types of WW domains, there is still redundancy regarding the presence of a specific type of WW domain in different proteins, and thus it is expected that upon targeting the interaction of a specific WW domain-containing protein, the interactions of different other WW domain-containing proteins will be affected. In this context, here, another issue is present, which is the presence of usually more than one WW domain in one protein. These domains do not seem to be redundant in their interactions. On the contrary, they seem to be very specific which raises another question; Which domain in a specific protein to target? Regarding the last possibility of targeting WW domain-containing protein partners as means of therapeutic intervention, it might seem to be the best option. Although the fact that different WW domain-containing proteins can partner with common and shared partners, there are different examples for the presence of specific targets for different WW domain-containing proteins. These specific targets might act as biomarkers to predict specific treatment success or even be a therapeutic target.

Finally, it is obvious that WW domain-containing proteins are critical factors in breast carcinogenesis and that a better understanding of their roles in breast cancer will likely lead to the identification and development of biomarkers and drug targets for cancer treatment.

## Author Contributions

AJ prepared figures and bibliography. ZS wrote the manuscript.

### Conflict of Interest Statement

The authors declare that the research was conducted in the absence of any commercial or financial relationships that could be construed as a potential conflict of interest.
